# Einfluss des COVID-19-Shutdowns auf die Arbeitsleistung einer Universitäts-Augenpoliklinik

**DOI:** 10.1007/s00347-021-01374-9

**Published:** 2021-04-21

**Authors:** C. Framme, J. Gottschling, P. Buley, K. Rohwer-Mensching, B. Junker, M. Dittberner, I. Volkmann

**Affiliations:** 1 Universitäts-Augenklinik, Medizinische Hochschule Hannover (MHH), Carl-Neuberg-Str. 1, 30652 Hannover, Deutschland; 2 Stabsstelle PM2 Klinische Leistungsentwicklung, Medizinische Hochschule Hannover, Carl-Neuberg-Str. 1, 30652 Hannover, Deutschland; 3 Zentrum für Informationsmanagement (ZIMT), Medizinische Hochschule Hannover, Carl-Neuberg-Str. 1, 30652 Hannover, Deutschland

**Keywords:** Patientenmanagement, Zeiterfassung, Sars-CoV-2, Poliklinik, Augenheilkunde, Patient management, Time measurement, Sars-CoV-2, Outpatient care, Ophthalmology

## Abstract

**Hintergrund:**

Die Coronaviruserkrankung COVID-19 hat im Frühjahr 2020 zu einer deutlichen Minderleistung der elektiven Medizin in den Krankenhäusern geführt, wobei es für universitäre Polikliniken bisher keine entsprechenden Daten über das Ausmaß dieser Reduktion und die damit verbundenen Erlösminderungen gibt.

**Material und Methode:**

Mithilfe der Daten des aus dem Krankenhausinformationssystems (IS-H/i.s.h.med unter SAP, Cerner Corporation, North Kansas City, MO, Vereinigte Staaten von Amerika und SAP SE, Walldorf, Deutschland) und der an unserer Klinik mitentwickelten Zeiterfassungs- und Managementsoftware TimeElement (Medizinische Hochschule Hannover, Hannover, Deutschland) wurden alle Patientenkontakte des COVID-19-Shutdowns über ca. 7 Wochen vom 18.03.2020 bis zum 08.05.2020 evaluiert und mit dem Vorjahreszeitraum 2019 verglichen. Zudem wurden die Fallzahlen für das erste und zweite Quartal 2019 und 2020 in Relation gesetzt.

**Ergebnisse:**

Im COVID-19-Zeitraum reduzierte sich die Gesamtzahl der Patientenkontakte um 59,5 % gegenüber dem Vorjahr. Die Anzahl der abrechenbaren Fälle reduzierte sich um 74,8 %.

Insbesondere der Hochschulambulanz‑/Selbstzahlerbereich verzeichnete mit einer Reduktion der Patientenkontakte auf 17,2 % des Ausgangswertes von 2019 den größten Patientenwegfall. Aus der reduzierten Arbeitsleistung resultierte ein Erlösverlust von mindestens 218.000 €. Über TimeElement ergab sich ein Rückgang aller diagnostischen Spezialleistungen von 69,4 %, wobei gerade auch Gesichtsfelduntersuchungen um ca. 75,3 % reduziert waren. OCT-Messungen verzeichneten einen Rückgang um 60,3 %. Das Patiententracking ergab allerdings auch eine Reduktion der durchschnittlichen Anwesenheitszeiten der Patienten um ca. 23 % (COVID-19: 145,8 ± 88,8 min vs. 2019: 189,6 ± 97,2 min).

**Diskussion:**

Der COVID-19-Shutdown ließ die Arbeitsleistung unserer Poliklinik auf nur noch ca. 40 % der Patientenkontakte und die der funktionsdiagnostischen Untersuchungen auf nur noch ca. 30 %, verglichen zur Leistung aus dem Jahr 2019, einbrechen. Die Reduktion der Patientenzahl führte allerdings auch dazu, dass die Anwesenheitszeiten der Patienten deutlich geringer als bei regulärer Auslastung ausfielen. Die damit verbundenen finanziellen Verluste sind durchaus erheblich und offensichtlich nicht über gesetzlich geregelte Ausgleichszahlungen wie im stationären Bereich kompensiert.

## Abstract (en)

**Background:**

The outbreak of the novel coronavirus disease 2019 (COVID-19) has led to a significant reduction in the number of patients treated in elective medicine in hospitals in spring 2020. Up until now, there are no corresponding data for university policlinics.

**Material and methods:**

With the help of the data of the clinic’s electronic medical record system (IS-H/i.s.h.med from SAP, Cerner Corporation, North Kansas City, MO, United States of America and SAP SE, Walldorf, Germany) and the time recording and management software TimeElement (Hannover Medical School, Hannover, Germany), which was codeveloped at our clinic, all patient contacts of the COVID-19 shutdown were evaluated over a period of approximately 7 weeks from 18 March 2020 to 8 May 2020 and compared with the same period in 2019. In addition, quarterly revenues for the first and second quarters of 2019 and 2020 were put into perspective via SAP.

**Results:**

During the COVID-19 period the total number of patient contacts was reduced by 59.5% compared to the previous year. The number of lockdown period cases decreased by 74.8% compared to the same period in 2019. In particular, the university outpatient/self-paying sector recorded the largest number of lost patient contacts with a reduction to 17.2% of the 2019 baseline. The reduced patient contacts and case numbers resulted in a significant financial loss of at least € 218,000.

Via TimeElement there was a 69.4% decrease in all special diagnostic services, with visual field examinations in particular also reduced by 75.3%. Optical coherence tomography (OCT) measurements recorded a decrease of 60.3%. Patient tracking revealed a reduction of average patient attendance times by approximately 23% (COVID 19: 145.8 ± 88.8 min versus 189.6 ± 97.2 min in 2019).

**Discussion:**

The COVID-19 shutdown caused the work performance of our policlinic to drop to only about 40% of patient contacts and that of functional diagnostic examinations to only about 30%, compared to the performance in 2019; however, the reduction in the number of patients also led to a significant reduction in the time patients were present at the clinic compared to the regular workload. The associated financial losses are considerable and obviously not compensated by legally regulated compensation payments as in the inpatient sector.

Laut niedersächsischem Beschluss vom 18.03.2020 [11] wurde ein sog. „Shutdown“ aufgrund der COVID-19-Pandemie in den Krankenhäusern ab dem 18.03.2020 verfügt, der schließlich über ca. 7 Wochen bis zum 06.05.2020 andauerte [12]. Diesbezüglich war es sowohl stationär als auch ambulant nur noch erlaubt, medizinisch dringend indizierte Leistungen zu erbringen. Eine entsprechende Definition solcher Leistungen fand nicht statt, sondern es wurde vielmehr den Kliniken überlassen, diese Leistungen sinnvoll und gewissenhaft festzulegen.

Vonseiten der Deutschen Ophthalmologischen Gesellschaft (DOG), der Retinologischen Gesellschaft (RG), dem Verband Deutscher Ophthalmologischer Chefärzte (DOCH) sowie der Vereinigung Ophthalmologischer Lehrstuhlinhaber (VOL) wurde im März 2020 eine Aufstellung erarbeitet, welche die Leistungen in dringende und weniger dringende Maßnahmen respektive elektive Fälle klassifizierte [8]. Demnach gibt es klar definierte ophthalmologische Notfälle wie etwa retinale Gefäßverschlüsse, Glaukomanfälle bzw. dekompensierte Glaukome, Erkrankungen mit Augenschmerzen, Endophthalmitiden und Traumata. Weiterhin sollten aber auch dringliche postoperative Kontrollen sowie chronische Therapien wie die intravitrealen operativen Medikamenteneingaben (IVOMs) inklusive Kontrollen durchgeführt werden, um eine Verschlechterung des Krankheitszustandes idealerweise zu verhindern. Auch onkologische Erkrankungen sollten innerhalb dieser Zeit kurzfristig behandelt werden. Zu den elektiven und nicht mehr durchzuführenden Behandlungen gehören neben der refraktiven Chirurgie natürlich auch die weitläufig durchgeführte Kataraktchirurgie inklusive Voruntersuchungen sowie andere aufschiebbare Behandlungen. Auch international wurden verschiedene Empfehlungen bezüglich dringender ophthalmologischer Behandlungen publiziert [1, 9, 10, 15].

Diese besondere Gesetzeslage führte dazu, dass innerhalb kürzester Zeit ein Großteil von Terminpatienten mit „elektiven“ Behandlungen nicht mehr einbestellt wurde und die Behandlung bis auf Weiteres ausgesetzt werden musste. Die politische Zusage einer finanziellen Kompensation von COVID-19-bedingten Mindereinnahmen in den Krankenhäusern schien dabei maßgeblich für den stationären Bereich mit freigehaltenen Betten zu gelten. Ein gesetzlich geregelter, finanzieller Ausgleich für den ambulanten Bereich fand bisher nicht statt.

Es stellt sich für uns die Frage, in welchem Maße die Arbeitsleistung in unserer Augenpoliklinik im besagten „COVID-19-Zeitraum“ zurückgegangen ist und wie – unabhängig davon, dass keine Patienten für spätere ggf. stationäre Operationen rekrutiert werden konnten („sekundärer Verlust“) – sich ggf. auch der direkte finanzielle Verlust für den ambulanten Bereich darstellt. In diesem Zusammenhang sei erwähnt, dass die COVID-19-Belastung der Krankenhäuser im Norden Deutschlands in der ersten Welle nur relativ moderat gewesen ist und somit viele Mitarbeiter über längere Zeiträume innerhalb des COVID-19-Shutdowns aufgrund ausgebliebener Patientenarbeit Überstunden abbauen konnten. Somit standen aber – wie in der regulären Wirtschaft auch – den fehlenden Einnahmen weiterlaufende Personalkosten gegenüber. Aus betriebstechnischer Sicht musste hier dem niedrigeren Patientenaufkommen entsprechend adäquat der Personalbedarf gesondert angepasst werden. Interessanterweise ist die inhaltliche Arbeitsleistung einer Augenpoliklinik außer im Bereich der Kontakte und der Fallanzahl unseres Wissens bisher nicht detailliert dargestellt. Somit gibt es unseres Wissens keine veröffentlichten Daten darüber, wie viel inhaltliche Arbeitsleistung beispielsweise in der Funktionsdiagnostik einer Universitäts-Poliklinik steckt. Diese mag wahrscheinlich zeitintensiver sein als in einer Praxis, da in einer Hochschulambulanz die meisten Patienten von fachärztlichen Kolleginnen überwiesen werden und somit die Universitätsklinik oft auch als weiterführende Instanz diagnostizieren und behandeln muss. Zusätzlich werden Assistenzärzte mittels 4‑Augen-Prinzip im Rahmen der oberärztlichen Patientenvorstellung/Supervision weitergebildet.

Über die in unserem Haus eingeführte Software „TimeElement“ ist es möglich, sämtliche Patienten der Augenpoliklinik virtuell online zu erfassen und den Patientenweg durch die verschiedenen Untersuchungsstationen innerhalb der Klinik nicht nur detailgetreu im Sinne eines „Patienten-Tracking“ zu verfolgen, sondern auch die internen Überweisungsaufträge in die unterschiedlichen Funktionseinheiten online per Knopfdruck zu generieren und im Zielgebiet abzurufen [6]. Damit sind wir in der Lage, den Patientenweg eines jeden einzelnen Patienten minutengenau online zu erfassen und idealerweise auch Untersuchungs‑/Wege- und Wartezeiten bei Erkennung freier Kapazitäten für einzelne Untersuchungen so gering wie möglich zu halten.

Ziel dieses Manuskriptes war es nun, die Evaluation der Leistungsentwicklung einer Augenpoliklinik unter den besonderen COVID-19-Bedingungen darzustellen. Hierbei liegt der Fokus sowohl auf der Abbildung der Anwesenheitszeit und des Patientenflows in den einzelnen Untersuchungsabschnitten sowie basierend auf den entsprechenden Fallzahlen auch auf den finanziellen Einbußen durch den Shutdown für das Gesamtkonzept Poliklinik.

## Material und Methoden

In Zusammenarbeit mit unserer administrativen Abteilungen wurde die für die Leistungsberechnung erforderliche Datenbasis aus den Datenbanken im IS-H/i.s.h.med (Cerner Corporation, North Kansas City, MO, Vereinigte Staaten von Amerika und SAP SE, Walldorf, Deutschland), dem COINS (Controlling-Informations-System, COINS Information Systems AG, Köln, Deutschland) und der in der Augenklinik zusätzlich genutzten Datenbank TimeElement (Medizinische Hochschule Hannover, Hannover, Deutschland) [6] extrahiert. Für den oben genannten Zeitraum des COVID-19-Shutdowns (37 Arbeitstage; 18.03.–08.05.2020 [inklusive 2 zusätzlicher Tage aufgrund notwendiger Vorbereitungszeit für Wiederaufnahme des Betriebes]) wurden über SAP sowohl für das Jahr 2020 als auch für den Vergleichszeitraum in 2019 (18.03.–08.05.2019) sämtliche Fallzahlen und Kontakte der Augenpoliklinik an regulären Arbeitstagen zur regulären Arbeitszeit von 7.30 bis 16.30 Uhr evaluiert (Notfälle außerhalb dieser Zeiten und am Wochenende wurden nicht betrachtet und aussortiert). Der Zeitraum ist hinsichtlich Arbeitstagen, Wochenenden und Feiertagen identisch. Durch die veränderten Wochentage ist in der Analyse für 2019 ein zusätzlicher Tag mit intravitrealen Injektionen enthalten.

Hinsichtlich der Erlöskalkulation wurden sämtliche Fallzahlen der ersten und zweiten Quartale 2019 und 2020 gesammelt und in Relation gesetzt. Die Daten zur Berechnungsgrundlage sind zudem in Tab. 1 aufgeführt.

**Table 1:** 

Grunddaten	2020	2019
Beginn der Messung	18.03.2020	18.03.2019
Ende der Messung	08.05.2020	08.05.2019
Tage	52	52
Werktage	35	35
Samstage, Sonntage, Feiertage	17	17
IVOM-Tage	15	16
Zeitfenster für die Auswertung Alle Patienten und Fälle innerhalb dieses Zeitraumes werden gezählt	Mo–Do 07:30–16:30 Fr 07:30–16:00	Mo–Do 07:30–16:30 Fr 07:30–16:00

In der Regel werden nahezu alle externen Patienten ambulant über einen Facharzt für Augenheilkunde unserer Ambulanz zugewiesen. Notfallpatienten werden zu den regulären Sprechzeiten ebenfalls im Rahmen der Hochschulambulanz behandelt.

Regelhaft werden die Patienten nach der administrativen Aufnahme in die Augenpoliklinik am Anmeldeschalter einem Assistenzarzt zugewiesen. Zuvor bekommen Neupatienten einen nichtärztlichen Vorabstatus mittels automatisierter Refraktionsbestimmung und automatisierter Sehschärfebestimmung (Topcon KR-800S, Tokyo Optikal Company Nippon, Itabashi, Japan) sowie einer Non-Contact-Luftdruckmessung des Augeninnendruckes (Nidek NT 510 noncontact tonometer, Nidek Co., Gamagori, Japan). Danach erhebt der Assistenzarzt die Anamnese und führt vor entsprechender medikamentöser Pupillenerweiterung sowohl die Vorderabschnittsuntersuchung an der Spaltlampe als auch orientierende weitere Untersuchungen wie Augenmotilitätsprüfungen, binokulares Sehen, Pupillenprüfungen etc. durch. Notwendige weiterführende Untersuchungen wie optische Kohärenztomographie (OCT), Fluoreszeinangiographie (FAG), Gesichtsfelduntersuchung (GF) etc. werden durch „interne Überweisung“ mittels TimeElement bei unserer Funktionsdiagnostik angemeldet. Schließlich erfolgt danach beim Assistenten die Untersuchung in Mydriasis mit entsprechender Fundoskopie gefolgt von der Oberarztvorstellung (Vier-Augen-Prinzip). Weiterführende Untersuchungen oder auch kleine Eingriffe (Poli-OP) würden gleichermaßen angemeldet werden. Generell hat jeder Patient somit mindestens 2‑mal während seines Besuches Arztkontakt.

**Figure 1:**
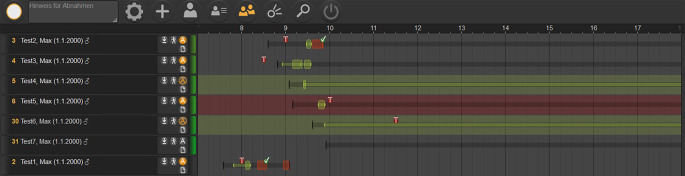

**Figure 2:**
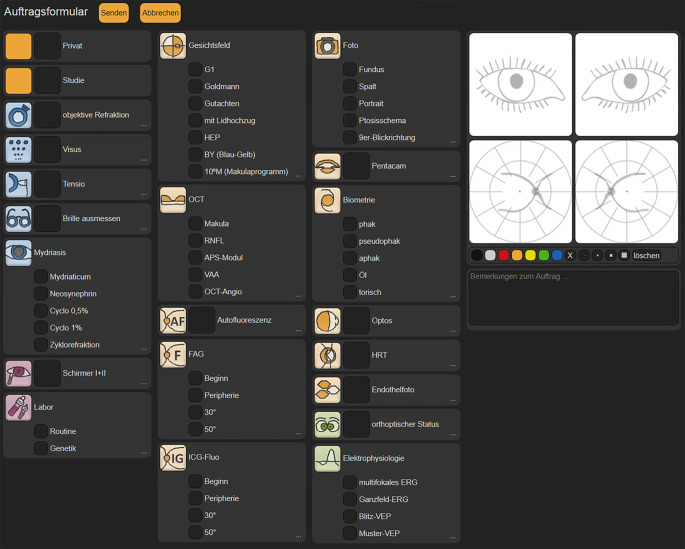

Erfolgte vor Einführung des TimeElement-Systems die Patientenführung über entsprechend ausgefüllte Wegezettel, ist über TimeElement jeder in der Poliklinik anwesende Patient im System erfasst und die aktuelle „Position“ innerhalb des oben beschriebenen Poliklinik-Parcours zeitlich immer minutengenau dargestellt (Abb. 1). Die „Überweisungen“ in die Funktionsdiagnostik erfolgen jeweils durch Mausklick, und auch nach Beendigung der Funktionsdienstleistung wird der Patient durch Mausklick wieder zurück zum Assistenten geschickt, der online in seiner Arbeitsliste sieht, dass der Patient „fertig“ ist und nun wieder von ihm weiterbehandelt werden kann (Abb. 2). Über ein Online-Rufsystem kann der zuständige Oberarzt die aktuelle Anzahl an Patienten in den einzelnen Untersuchungskabinen auf seinem Endgerät einsehen, welche zur Supervision/Abnahme vorbereitet sind [6].

Bezüglich der Arbeitsleistung und der Abrechnungsmodalitäten sind die Begriffe Fall und Patientenkontakt voneinander zu unterscheiden. Der Fallbegriff beschreibt den Abrechnungsfall pro Quartal, egal wie oft der Patient gesehen wird. Diesen Begriff verwenden wir in unserer Auswertung für die Beschreibung der Erlöse und finanziellen Auswirkungen.

Patientenkontakte geben die exakten Patientenkontaktzahlen und Behandlungsereignisse in der Ambulanz wieder (auch beispielsweise mehrfach pro Quartal ohne weitere finanzielle Kompensation). Diese können sowohl aus SAP als auch aus TimeElement exportiert werden. Wir ziehen sowohl den Fall als auch die Patientenkontakte für die Auswertung der COVID-19-Reduktion auf das Geschehen in der Augenpoliklinik der MHH heran, inklusive des damit jeweils verbundenen Arbeitsaufwandes. In SAP werden die administrativen Daten (der Behandlungsfall pro Quartal und die Anwesenheit des Patienten in der Augenpoliklinik) erfasst, in TimeElement kommen noch konkrete Behandlungsdetails, die auch in einem wissenschaftlichen Kontext interessant sind, hinzu. Daher wurden die Daten aus beiden Datenpools, sowohl aus SAP als auch aus TimeElement, genutzt und für die vorliegende Betrachtung exportiert. Neben der Fallextraktion über SAP wurden für die zu untersuchenden Zeiträume auch alle im System TimeElement erfassten Patienten und Leistungen numerisch evaluiert, sowie auch die Gesamtanwesenheitszeit wurde überprüft. Dabei ist zu beachten, dass SAP sowohl Patientenfälle als auch Patientenkontakte erfasst, TimeElement hingegen nur die ambulanten Patientenkontakte lückenlos abdeckt. Dafür kann TimeElement unterschiedliche Prozesse innerhalb eines Besuchs abbilden. Einige Patienten (z. B. IVOM-, Studien- und Konsilpatienten) werden aus internen Organisationsgründen nicht regelhaft über TimeElement erfasst, sodass die Kontaktanzahl in der TimeElement-Auswertung niedriger ausfällt als diejenige aus der SAP-Datenbank (alle dokumentierten Poliklinik-Kontakte). Time Element wurde daher maßgeblich für die inhaltliche Arbeitsleistung des regulären Sprechstundenbereiches der Hochschulambulanz genutzt.

Nach Maßgabe der Fachgesellschaften für Behandlungsdringlichkeiten [8] wurden während des Shutdowns, wie oben beschrieben, nur noch dringend indizierte Behandlungen durchgeführt. Dieses beinhaltete in unserem Setting die Behandlung von Notfallpatienten, aber auch die weitere Durchführung der IVOMs bei chronischen Makulaerkrankungen. Ebenfalls wurden ambulant dringende postoperative Kontrollen zur Evaluation des Operationsergebnisses durchgeführt. Sämtliche elektiven Termine wurden storniert.

Die der Auswertung zugrunde liegenden Daten sind in Tab. 1 aufgeführt. Die Auswertung der einzelnen Parameter erfolgte mittels Microsoft Excel 2016 (Microsoft Corporation, Redmond, Washington, Vereinigte Staaten).

## Ergebnisse

Innerhalb des Zeitraumes der COVID-19-bedingten Reduktion auf nur noch dringend medizinisch bedingte Behandlungen wurden laut SAP-Dokumentation während der regulären Arbeitszeit *n* = 1332 Patientenkontakte gezählt. Davon entfielen *n* = 351 auf die Hochschulambulanz-Behandlung (HSA inklusive Selbstzahlerpatienten [SZ]), *n* = 89 auf das ambulante Operieren (AO), *n* = 507 auf die IVOMs (IVOMs über HSA-Pauschale, BDOC-Verträge und Privatliquidation [PL]/SZ) sowie *n* = 385 auf die internen Konsilleistungen (KO).

Im Vergleichszeitraum 2019 ergaben sich insgesamt *n* = 3291 Patientenkontakte; wovon im HSA/SZ-Bereich 2035 Kontakte, *n* = 224 Kontakte im Bereich AO, *n* = 532 Kontakte im IVOM-Bereich (aufgrund der Wochentage 2019 mit einem zusätzlichen Tag IVOMs) sowie im Bereich KO *n* = 500 Kontakte stattfanden (Tab. 2).

**Table 2:** 

	2020	2019	Veränderung 2020 im Vergleich zu 2019 (%)
Fälle Q1 + Q2	4616	6064	−23,9
HSA/SZ Q1 + Q2	3285	4345	−24,4
SAP-Fälle (Lockdown-Zeitraum)	527	2092	−74,8
SAP-Kontakte	1332	3291	−59,5
Hochschulambulanz (HSA) und Selbstzahler (SZ)	351	2035	−82,2
IVOMs	507	532	−4,7
Ambulantes Operieren (AO)	89	224	−60,3
Konsile	385	500	−23,0
TimeElement-Kontakte	1230	3161	−61,1
TimeElement-Kontakte/Tag	35,14	90,31	−61,1
Diagnostikaufträge	2126	6938	−69,4
Durchgeführte Autoref/NCT	241	694	−65,3
Durchgeführte Gesichtsfelder	175	708	−75,3
Durchgeführte OCT	661	1663	−60,3
Durchgeführte FAG	58	136	−57,4
Diagnostikaufträge/Patient	1,60	2,11	−24,3
Autoref/NCT/Patient	0,18	0,21	−14,2
Gesichtsfelder/Patient	0,13	0,22	−38,9
OCT/Patient	0,50	0,51	−1,8
FAG/Patient	0,04	0,04	5,4
Interaktionen Diagnostik	1,99	2,31	−13,9
Patientenanwesenheit (Mittelwert/Median in min)	145,8/127,2 ±88,8	189,6/182,4 ±97,2	−23,1/−30,3
Zeit für ärztliche Interaktion (Mittelwert/Median in min)	11,6/9,6 ±9,62	11,4/9,4 ±9,38	1,8/2,1
Zeit für Funktionsdiagnostik (Mittelwert/Median in min)	10,5/7,1 ±9,50	8,8/5,7 ±8,47	19,3/24,6
Wartezeit (Mittelwert/Median in min)	102,9/85,7 ±78,1	137,5/131,3 ±78,9	−25,1/−34,7

*HSA* Hochschulambulanz, *SZ* Selbstzahler, *IVOMs* Intravitreale Injektionen, *AO* Ambulantes Operieren, *Autoref* Autorefraktion, *NCT* Non-Contact-Tonometrie, *OCT* Optische Kohärenztomographie, *FAG* Fluoreszenzangiographie

**Figure 3:**
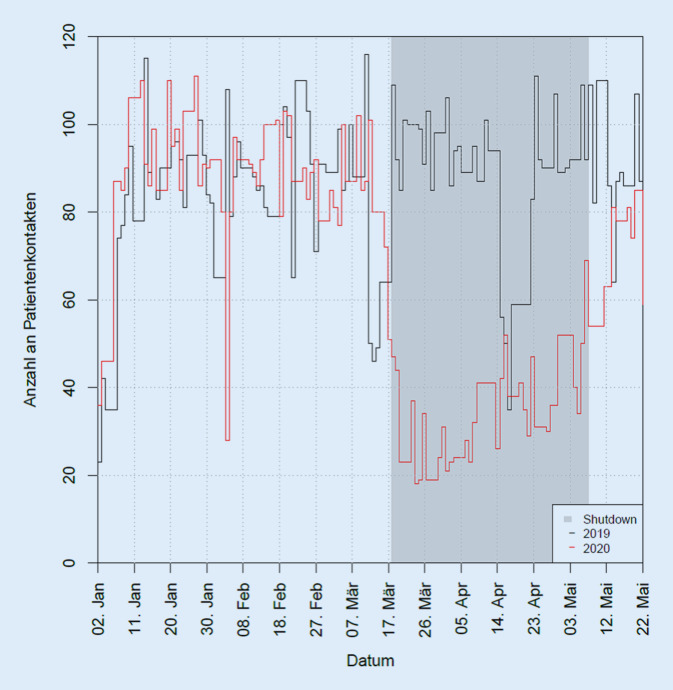

Somit reduzierte sich durch COVID-19 die Leistung unserer Poliklinik gemessen an der Anzahl der Patientenkontakte auf nur noch 40,5 % der Ursprungsleistung aus dem Jahr 2019 (Reduktion in HSA/SZ auf 17,2 %; in AO auf 39,7 %, in IVOM auf 95,3 % und in KO auf 77,0 %). Die Abb. 3 und 4 zeigen anschaulich die Reduktion der Patientenzahlen des Jahres 2020 gegenüber 2019 im COVID-19-Zeitraum (Abb. 3 und 4).

**Figure 4:**
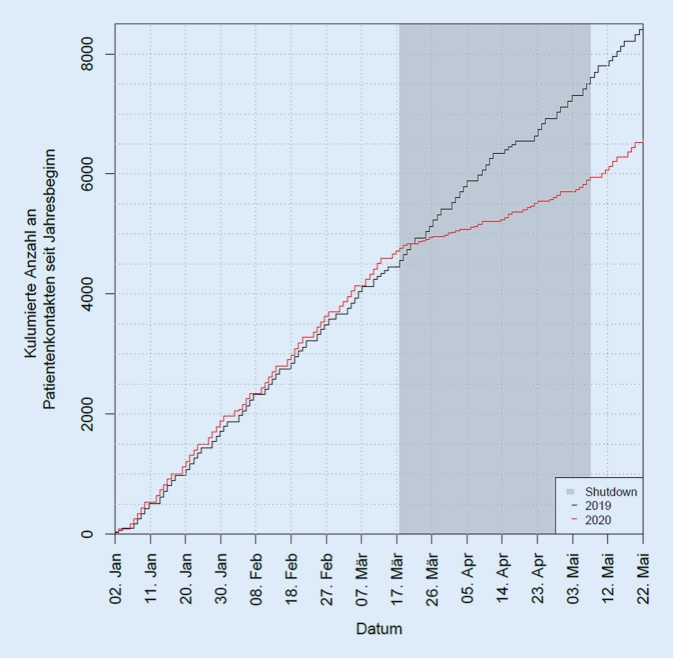

Unter Betrachtung der Quartalszahlen ergibt sich eine Reduktion der Fälle (ein Abrechnungsfall kann mehrere Kontakte in einem Quartal enthalten!) von 6064 auf 4616 Fälle (−1448 Fälle, −23,9 %) für die ersten beiden Quartale. In der Betrachtung der einzelnen Quartale zeigt sich für Quartal 1 eine Reduktion von 3079 auf 2728 Fälle (−351 Fälle, −11,4 %) sowie für Quartal 2 eine Reduktion von 2985 auf 1888 Fälle (−1097 Fälle, −36,8 %).

Für die Zeit des Lockdowns ergab sich bezüglich der abrechenbaren HSA/SZ-*Fälle* (alle über HSA abrechenbaren ambulanten Fälle inklusive IVOMs) eine Reduktion von 2092 auf 527 Fälle (minus 1565 Fälle; entspricht −74,8 %). Bei einer HSA-Pauschale an der Augenpoliklinik in 2019 und 2020 von 145 €/Quartal betrug der COVID-19-bedingte Umsatzverlust für diesen Zeitraum somit mindestens 226.925 € für den HSA/SZ-Bereich der Poliklinik (HA-Anteil 88,4 %; SZ-Anteil 11,6 %). Unter der Berücksichtigung, dass 85 % aller Fälle (interne Auswertung der Fallbesuche pro Quartal in unserer Klinik in 2019) nur einen Besuch pro Quartal aufweisen, könnte trotz Verlust während der Lockdown-Zeit der Fall im restlichen Quartal in somit ca. 15 % dennoch erlöst werden. Somit könnte der reale finanzielle Verlust überschlagsweise noch auf mindestens aber ca. 193.000 € in diesem Bereich reduziert sein.

Die Reduktion der Privatleistungen sowie die Reduktion im Bereich AO führen zu weiteren Verlusten. Alleine der AO-Anteil führt zu einem weiteren direkten Verlust von ca. 25.000 € (Erlösverlust der Leistungsstelle ambulantes Operieren für oben genannten Zeitraum). Somit darf insgesamt von einem geschätzten direkten Verlust von ca. 250.000 € für alle ambulanten Leistungen unserer Poliklinik ausgegangen werden.

**Figure 5:**
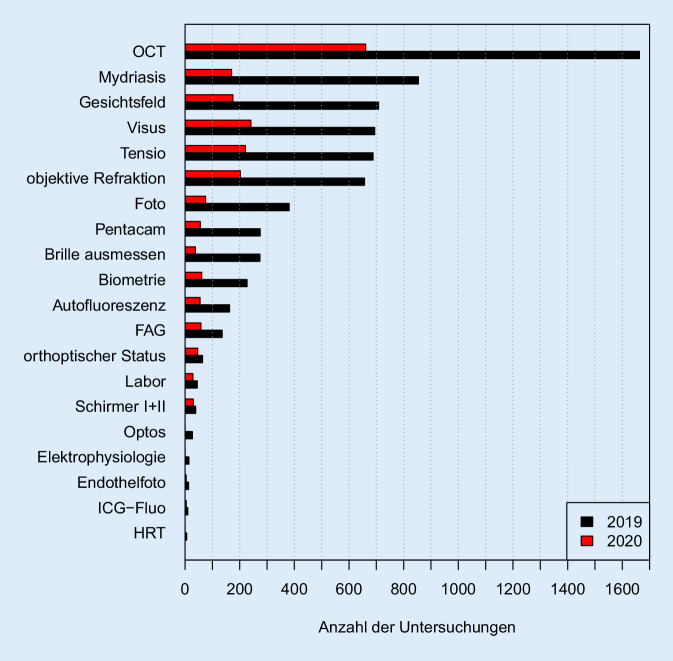

Mittels unserer Poliklinik-eigenen Software TimeElement wurden im COVID-19-Zeitraum 2020 insgesamt 1230 Kontakte und im Jahr 2019 insgesamt 3161 Kontakte aufgezeichnet (Rückgang 61,1 %). Hier zeigt sich eine relativ gute Übereinstimmung zu den Kontaktzahlen, die aus SAP extrahiert werden konnten (s. oben). Bezogen auf die Kontakte pro Tag ergab sich eine signifikante Reduktion von durchschnittlich 90,31 Kontakten in 2019 auf 35,14 Kontakte in 2020. Der mit dieser Kontaktreduktion einhergehende Rückgang der einzelnen Diagnostikarten zeigt Abb. 5. Durchschnittlich errechnete sich ein Rückgang von 69,4 % (*n* = 2126/6938) über alle aufgeführten funktionsdiagnostischen Untersuchungen. Die Rückgänge der prägnantesten Untersuchungen betrugen für jeweils 2020 vs. 2019: A) OCT: *n* = 661/1663; −60,3 %; B) Visus/Tensiomessung: *n* = 241/694; −65,7 %; C) GF: 175/708; −75,3 %; D) FAG: 58/136; −57,4 %.

**Figure 6:**
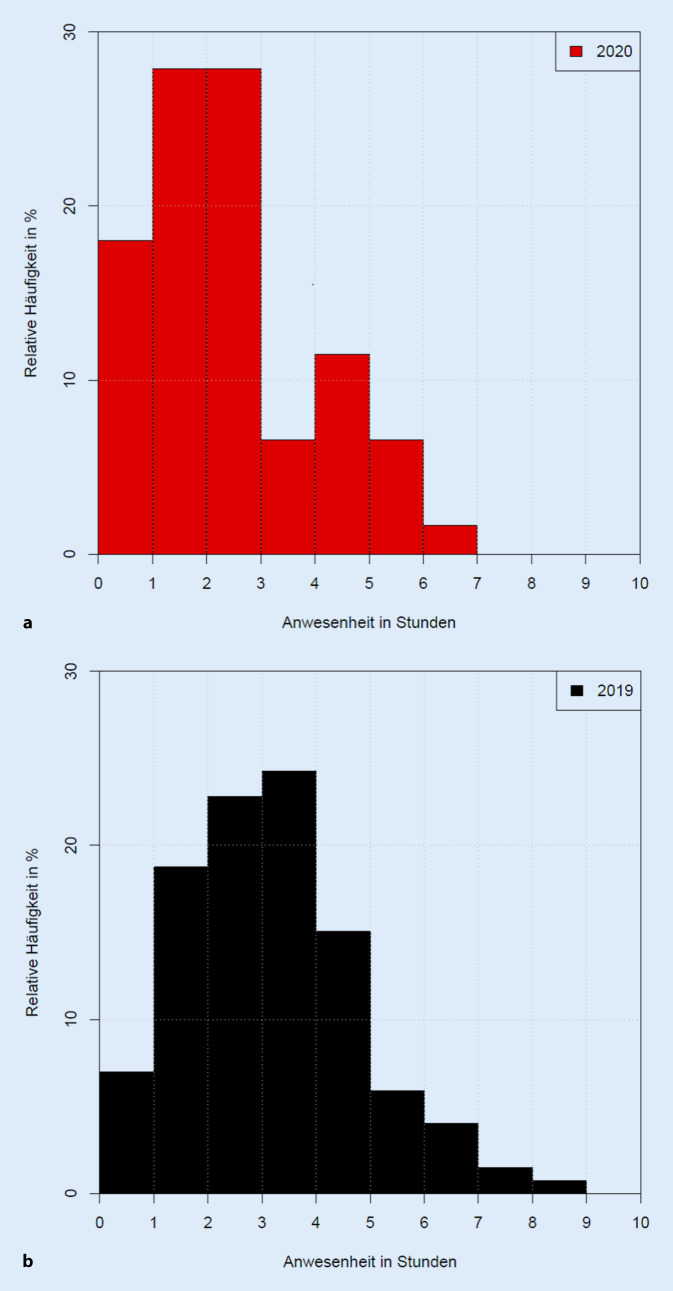

Das zeitliche Patiententracking über TimeElement ergab folgende durchschnittliche Anwesenheitszeiten (Mittelwert/Median ± Standardabweichung) für 2020: 145,8/127,2 ± 88,8 min vs. 2019: 189,6/182,4 ± 97,2 min. Die gesamte Anwesenheitszeit hat sich somit durchschnittlich um 23,1 % verkürzt, und Patienten wurden im COVID-19-Zeitraum schneller behandelt (Abb. 6). Diese schnellere Behandlung war prinzipiell verkürzten Wartezeiten zwischen den einzelnen Untersuchungspunkten zuzuordnen (2020: 102,9/85,7 ± 78,1 min vs. 2019: 137,5/131,3 ± 78,9 min). In der Wartezeit enthalten sind messtechnisch nicht näher erfasste Schritte wie Wegzeiten, Wartezeit für medikamentöse Mydriasis und Ähnliches. Die ärztliche Interaktion mit einem Patienten (jeweils mindestens 2‑mal pro Patient) während des COVID-19-Zeitraums dauerte prinzipiell ähnlich lange wie im Vergleichszeitraum (2020: 11,6/9,6 ± 9,62 min vs. 2019: 11,4/9,4 ± 9,38 min). Funktionsdiagnostische Untersuchungen beanspruchten pro Interaktion durchschnittlich 10,5/7,1 ± 9,50 min (2020) vs. 8,8/5,7± 8,47 min (2019). In der Regel fanden pro Patient im Median jeweils 2 relevante Interaktionen sowohl in der Diagnostik (z. B. OCT, FAG, GF, Visus/Tensio) als auch mit dem ärztlichen Personal (assistenzärztliche Voruntersuchung sowie oberärztliche Supervision) statt. Durchschnittlich war hier eine Reduktion von 2,31 auf 1,99 Interaktionen in der Diagnostik erkennbar, was eine auch inhaltlich etwas reduzierte Leistung pro Patient unter COVID-19 anzeigt, wobei allerdings wesentliche Untersuchungen wie FAG und OCT eine Korrelation zur reduzierten Patientenanzahl aufweisen (0,5 OCT/Patientenkontakt 2020 vs. 0,51 OCT/Patientenkontakt 2019 sowie 0,04 FAG/Patientenkontakt 2020 vs. 0,04 FAG/Patientenkontakt 2019).

## Diskussion

Mit dem Auftreten von COVID-19 hat sich schlagartig eine neue Normalität nicht nur im realen Leben, sondern gerade auch im Krankenhaus eingestellt. War man im Frühjahr 2020 von den in den Medien weitverbreiteten Bildern aus Italien und Spanien sowie anderen Ländern mit den vielen Todesfällen gewarnt, hat man in Deutschland relativ frühzeitig zu Beginn der COVID-19-Welle einen sog. „Shutdown“ des öffentlichen Lebens mit entsprechenden Abstandsregeln erlassen [3]. Krankenhäuser waren zügig gezwungen, das gesamte elektive Behandlungsprogramm zu stornieren, insbesondere um ausreichend Kapazitäten auf den Intensivstationen mit den entsprechenden Beatmungsmöglichkeiten freizuhalten. Im gleichen Rahmen bedeutete das aber auch einen Stopp für ambulante Elektivbehandlungen, die an sich zumindest im Rahmen der universitären Augenheilkunde keine Intensivkapazitäten binden. Dieses führte dazu, dass in Niedersachsen der „Elektiv-Stopp“ vom 18.03.2020 bis zum 06.05.2020 beschlossen wurde [11, 12] und in unserer Augenpoliklinik bis zum 08.05.2020 nur noch dringend medizinisch indizierte Leistungen erbracht wurden. Die ophthalmologischen Aspekte der COVID-19-Pandemie wurden in einem Übersichtsartikel aus der Augenklinik der LMU in München bereits detailliert dargestellt [14]. Das Interesse unseres Manuskriptes ist es, die primären Auswirkungen des COVID-19-Shutdowns insbesondere auf die finanziellen Belange, die Arbeitsleistung bezüglich Patientenanzahl und der detaillierten Leistungen des ärztlichen und nichtärztlichen Personals unserer Poliklinik zu erfassen. Über die Software TimeElement [6], welche bundesweit lediglich in unserer Poliklinik standardisiert angewendet wird, ist es möglich, den Patientenfluss digitalisiert darzustellen und personengenau alle Arbeitsschritte, Arbeitszeiten sowie Wartezeiten zu erfassen. Somit kann gegenüber dem Vorjahr eine exakte detailgetreue Aufstellung des Arbeitsaufkommens ermöglicht werden.

Die Ergebnisse zeigen nun auf Basis zweier verschiedener Datenbanksysteme (SAP und TimeElement), dass die Gesamtkontakte in der Poliklinik um 59,5 % eingebrochen sind, wobei der am stärksten frequentierte Bereich der reinen Hochschulambulanz inklusive Selbstzahlern um ca. 82,8 % eingebrochen ist.

Da auf berufspolitischer Ebene frühzeitig entschieden wurde, dass es auch im COVID-19-Zeitraum essenziell wichtig ist, die IVOMs zur chronischen Therapie und Stabilisierung von Makulaerkrankungen weiterzuführen [8], war die Reduktion der Patientenkontakte in diesem Segment mit etwa 4,7 % in unserem Kollektiv gering. Da in unserer Klinik die IVOMs aber hauptsächlich am Dienstag und Mittwoch durchgeführt werden und im direkten Vergleich der COVID-19-Zeiträume durch eine 2‑Tage-Verschiebung ein IVOM-Tag weniger in 2020 (bei gleicher Anzahl Arbeitstage: *n* = 35) ausgewertet wurde, darf davon ausgegangen werden, dass die IVOM-Zahlen konstant gewesen sind. Nichtsdestotrotz sind aber auch einige Patienten aus Angst vor Ansteckung der Klinik ferngeblieben und tun dieses auch noch weiterhin. Diese nicht unerhebliche Thematik des Ausbleibens von Patienten mit entsprechender Behandlungsdringlichkeit aus Angst vor Ansteckung wurde ebenfalls ausführlich im *Deutschen Ärzteblatt* diskutiert [4].

Der Bereich ambulantes Operieren (AO) reduzierte sich um 60,3 %, sodass zumindest die knappe Hälfte der Operationen noch durchgeführt werden konnte. Das liegt daran, dass die Tumorchirurgie im Lidbereich auf Basis nicht langfristig zu verschiebender Onkologie ebenfalls in Abhängigkeit der Diagnosedringlichkeit partiell weitergeführt werden musste. Hausinterne Konsilleistungen wurden in immerhin noch fast 80 % weitergeführt, was anzeigt, dass der Bereich der stationären Bettenbelegung mit entsprechenden Konsilfragestellungen keiner so großen Reduktion unterlag wie der ambulante Bereich, da gesamthaft immer noch der überwiegende Teil aller Betten (unabhängig von den Intensivstationen) belegt war (Reduktion der klinikinternen, nichtaugenheilkundlichen Betten im COVID-19-Zeitraum: 28 %).

Dem gegenüber steht die Betrachtung der Behandlungsfälle. Diese sind auf den Lockdown-Zeitraum betrachtet um 75 % im Vergleich zum Vorjahr eingebrochen. Unter Berücksichtigung des gesamten Abrechnungszeitraumes der beiden betroffenen Quartale 1 und 2 haben sich die Fallzahlen um 24 % reduziert. Die absolute Reduktion der Fallzahlen ist dabei für Lockdown-Zeitraum (−1565 Fälle) und Abrechnungszeitraum (−1448 Fälle) ähnlich. Es scheint somit, dass die übrigen Behandlungstage durch den Lockdown nicht beeinträchtigt wurden und sogar einen Teil der ausgebliebenen Fälle kompensieren konnten (117 Fälle zusätzlich).

Es ist für die Erlösberechnung zu beachten, dass die oben genannten Fälle des Lockdown-Zeitraums auch theoretisch mehr als 1 Besuch pro Quartal aufweisen können und somit die Fallpauschale außerhalb des Lockdowns verdient werden kann. Interne Zahlen belegen, dass 85 % aller Fälle nur 1 Besuch pro Quartal aufweisen. Entsprechend haben wir die Berechnung der Umsatzverluste um diesen Wert reduziert (−1565 absolute Fälle, −1330 einfache Fälle nach Korrektur).

Der direkte finanzielle Verlust im Bereich unserer Poliklinik durch nicht erbrachte Leistungen beläuft sich auf mindestens ca. 218.000 € für den COVID-19-Zeitraum plus privater Leistungen, wobei hier einerseits die Personalkosten (mit partiellem Überstundenabbau, es wurden keine Mitarbeiter abteilungsfremd eingesetzt) weitergelaufen sind und anderseits die sekundären Verluste im stationären Bereich aufgrund der nahezu 7 Wochen fehlenden Rekrutierung von elektiven stationären Patienten für entsprechende Operationen nicht abzuschätzen sind. Diese Zahlen belegen deutlich, dass auch im ambulanten Poliklinikbereich sechsstellige Verluste in einer bei uns vergleichbar mittelgroßen Poliklinik mit im Jahresdurchschnitt etwa 22.000 Patientenkontakten (ca. 18.650 Fälle) durch COVID-19 zustande gekommen sind. Für die bereits ohnehin stark im Spannungsfeld der Ökonomie und Medizin agierenden Universitätskliniken ist dieser Zustand mehr als problematisch, und der weitere Verlauf der bisherigen Krankenhausfinanzierung darf gespannt abgewartet werden. Weiterhin ist auch unklar, ob spätere COVID-19-Ereignisse erneut einen Shutdown der Klinik nach sich ziehen können.

Augenpolikliniken sind in der Regel hoch spezialisierte Ambulanzen, welche maßgeblich auf Überweisung aus dem augenfachärztlichen Bereich agieren. Somit liegt es nahe, dass eine über das Normalmaß hinausgehende große diagnostische Bandbreite angeboten werden muss. Dabei fällt auf, dass im Gegensatz zum stationären Klinikbereich offensichtlich noch keine standardisierten und regelmäßigen Kostenträgerrechnungen der Leistungen im ambulanten Bereich vorhanden sind und auch keine wirkliche Vorstellung über die reale Arbeitsleistung insbesondere der funktionsdiagnostischen Einheiten in einer typischen Universitäts-Augenklinik existiert. Diese Lücke gilt es, im Sinne einer auskömmlichen Finanzierung zukünftig zu schließen, wenn die Ambulantisierung weiter voranschreitet.

Durch die Vorhaltung einer entsprechenden Anzahl ärztlicher und nichtärztlicher Mitarbeiter mit entsprechenden Personalkosten haben die COVID-19-bedingte Reduktion der Arbeitsleistung und der folgliche Einnahmenverlust gerade auch in den Ambulanzen einer Klinik – wie oben beschrieben – zu einem deutlichen Defizit beigetragen. Die in Abb. 5 dargestellte Auflistung der diagnostischen Maßnahmen, die in der Regel von nichtärztlichem Personal übernommen werden und neben der ärztlichen Leistung einen erheblichen Anteil in einer universitären Augenpoliklinik einnehmen, ergab im COVID-19-Zeitraum einen erheblichen Rückgang von ca. 70 %. Dennoch wurden im direkten Zahlenvergleich offensichtlich – und trotz leichter Reduktion der Gesamtanzahl diagnostischer Interaktionen – weiterhin alle relevanten Funktionsdiagnostiken bei den anwesenden Patienten adäquat durchgeführt (z. B. OCT: Reduktion der Leistung um ca. 60 % entspricht proportional der Patientenzahlreduktion).

Interessant erschien weiterhin der Fakt, dass zwar neben einer ähnlich langen ärztlichen Konsultationszeit in den beiden Zeiträumen die durchschnittliche Dauer der funktionsdiagnostischen Untersuchungen während COVID-19 deutlich länger ausfiel. Wesentliche Gründe dafür sehen wir in den umfassenderen Hygienevorschriften und Erläuterungen vor jeder Untersuchung. Während weitreichendere Hygienevorkehrungen bei der ärztlichen Untersuchung im Vorfeld jedes einzelnen direkten Arzt-Patienten-Kontaktes durchgeführt werden (und somit nicht zeitlich über TimeElement für diese Aktion erfasst sind), ist diese Zeit bei den Funktionsuntersuchungen mit inkludiert. Somit ist es uns in dieser Auswertung auch möglich, zumindest partiell den Mehraufwand für COVID-19-Hygiene zu dokumentieren, für welche die Gebührenordnung für Ärzte (GOÄ) seit 05.05.2020 eine neue Hygienepauschale von 14,75 € für Mehraufwand vorsah [16]. Diese Pauschale ist sowohl für den niedergelassenen Bereich als auch für Hochschulambulanzen abrechenbar und wurde ab dem 01.10.2020 auf 6,41 € reduziert.

Ein für die Patientenseite positiver Aspekt des COVID-19-Shutdowns war die gerade auch im Median gemessene deutliche Reduktion der Anwesenheitszeiten der Patienten in unserer Poliklinik, was natürlich insbesondere im Sinne einer Kontaktreduktion auf ein mögliches Minimum wünschenswert ist (Jahr 2020 Gesamtanwesenheitszeit: 2 h 26 min vs. Jahr 2019: 3 h und 10 min; Tab. 2). Dennoch zeigen die mit immer noch deutlich über 2 h durchschnittlichen Anwesenheitszeiten auch unter COVID-19, dass dezidierte Diagnostik und Weiterbildung (4-Augen-Prinzip) sowie Erstellung eines Therapieplanes doch immer noch erhebliche Zeit benötigen, wobei aber regelmäßig der Anteil der Wartezeiten auf die einzelnen Untersuchungen überwiegt. Die hohen Standardabweichungen in den Anwesenheitszeiten unterstreichen dabei die Individualität und zum Teil hohe Komplexität der notwendigen Behandlungen. Die Anwesenheitszeiten verdeutlichen, dass bei einer Reduktion der Patientenzahl auch die Gesamtanwesenheitszeit der Patienten sinkt. Durch die ungewollt höheren personellen Ressourcen und Untersuchungsmöglichkeiten sinkt die Gesamtbehandlungszeit deutlich. Hierfür gilt es in Zukunft, Engpässe sowohl in personeller als auch struktureller Hinsicht zu erkennen, um Wartezeiten zu reduzieren. Die notwendige personelle Besetzung einer Augenklinik und die möglicherweise dafür in der Krankenhausfinanzierung nicht hinreichend berücksichtigte hohe und komplexe Arbeitsleistung einer augenheilkundlichen Poliklinik sind auch Thema eines Arbeitskreises der DOG für mögliche zukünftige Verbesserungen [13]. Zudem könnten z. B. im Sinne einer Kostenträgerrechnung – wie beispielsweise gerade für strabologische Operationen durchgeführt [5] – auch die Kosten, Arbeitszeiten und Erlöse einer Hochschulambulanz dezidiert berechnet werden.

Über die Kommission Sektorübergreifende Augenheilkunde vom Berufsverband der Augenärzte (BVA) und der Deutschen Ophthalmologischen Gesellschaft (DOG) sind erste Zahlen für Deutschland zur Versorgungssituation während des COVID-19-Shutdowns verfügbar. Diese wurden im Rahmen einer Mitgliederbefragung erhoben und zeigen eine deutliche Reduktion der nichtnotfallmäßigen Versorgung in den verschiedenen augenheilkundlichen Versorgungsformen. Diese Daten decken sich mit unseren Fallzahlen, die eine deutliche Reduktion der ambulanten Versorgung unter Beibehaltung der IVOM-Versorgung, dringlicher Operationen wie der Tumorchirurgie und Notfalleingriffen zeigen [7].

Aus dem Ausland ist bereits eine Veröffentlichung aus Indien zu dem dortigen Behandlungsstopp verfügbar. In dem Manuskript werden durchschnittliche Reduktionen von 96–98 % der Behandlungen angeführt. Auch wenn die Erkrankung COVID-19 die gleiche ist, spiegelt dieser harte Shutdown eine ganz andere Reaktion des indischen Systems wider. In unserem Gesundheitssystem war die Versorgung von chronischen Erkrankungen und akuten Notfällen weiterhin möglich, sodass für viele Fälle zumindest keine nachhaltig schädigende Unterversorgung entstanden ist [2].

## Fazit für die Praxis

- Schlussfolgernd zeigt diese Auswertung, dass der COVID-19-Shutdown zu einer erheblichen Minderleistung in unserer Augenpoliklinik mit auch entsprechend hohen finanziellen Verlusten geführt hat. Dargestellt ist ansatzweise auch die Komplexität einer Universitäts-Augenpoliklinik, in der viele zusätzliche Leistungen zum Zwecke der Diagnose- und Therapiefindung, die sonst weder zeitlich noch kostentechnisch regulär abgebildet sind, mittels TimeElement dargestellt werden können. Somit ist zeitlich sowohl der erhöhte Hygieneaufwand darstellbar gewesen als auch die deutlich geringere Anwesenheitszeit der Patienten im COVID-19-Zeitraum. Dieses lässt – wie oben bereits diskutiert – vermuten, dass in regulären Zeiten die personelle Besetzung nicht der eigentlichen Patientenlast entspricht, was dazu führt, dass Patienten eher längere Anwesenheitszeiten erdulden müssen, dafür aber im Sinne einer allumfassenden spezifizierten Diagnostik und Therapie (häufig alles an einem Tag!) behandelt werden können.
- Als Ausblick wäre es sinnvoll die Daten anderer Häuser zusammenzutragen, um sich ein einheitliches Bild zu den oben genannten Punkten zu verschaffen. Dieses würde Aussagen darüber ermöglichen, wie produktiv die in der Regel als defizitär angesehenen Polikliniken wirklich sind und auch wie viel Personal für die erwartete Arbeitsleistung einer Augenpoliklinik regelhaft budgetiert werden müsste, um patientengerecht arbeiten zu können. Aktuell wünscht man sich aber zunächst erst einmal den Weg aus der COVID-19-Zeit heraus zurück zur gewissen Normalität.
